# Safety and Efficacy of Personalized Cancer Vaccines in Combination With Immune Checkpoint Inhibitors in Cancer Treatment

**DOI:** 10.3389/fonc.2021.663264

**Published:** 2021-05-28

**Authors:** Juan-Yan Liao, Shuang Zhang

**Affiliations:** ^1^ Department of Biotherapy, Cancer Center, West China Hospital of Sichuan University, Chengdu, China; ^2^ Sichuan Clinical Research Center of Biotherapy, Chengdu, China

**Keywords:** personalized cancer vaccine, immune checkpoint inhibitor, combination therapy, neoantigen, immunotherapy

## Abstract

Cancer immunotherapy can induce sustained responses in patients with cancers in a broad range of tissues, however, these treatments require the optimized combined therapeutic strategies. Despite immune checkpoint inhibitors (ICIs) have lasting clinical benefit, researchers are trying to combine them with other treatment modalities, and among them the combination with personalized cancer vaccines is attractive. Neoantigens, arising from mutations in cancer cells, can elicit strong immune response without central tolerance and out-target effects, which is a truly personalized method. Growing studies show that the combination can elevate the antitumor efficacy with acceptable safety and minimal additional toxicity compared with single agent vaccine or ICI. Herein, we have searched these preclinical and clinical trials and summarized safety and efficacy of personalized cancer vaccines combined with ICIs in several malignancies. Meanwhile, we discuss the rationale of the combination and future challenges.

## Introduction

Neoantigen, an abnormal protein stemming from “non-synonymous mutation”, is specific to tumor cells (1). Neoantigens are non-self-peptides without central tolerance and off-target immune-toxicity, which are the main barriers of previous cancer vaccines and the primary obstacles in the development of personalized cancer therapy (2–4). Therefore, they are “perfect” targets with strong immunogenicity to elicit effective antitumor activity. Currently, a lot of preclinical and clinical trials have proven that neoantigen vaccines are personalized therapy and can activate hosts’ immune systems which then promote redirected T cells to kill tumor cells. However, the single use of neoantigen vaccines has a limited efficacy.

In 2013, immune therapy of cancer was regarded as the most breakthrough in Science. From then, immunotherapy became a major focus in cancer therapy, of which immune checkpoint inhibitors are the most promising and concerned topic (5). Immune checkpoint inhibitors such as anti-PD-1 and anti-CTLA-4 monoclonal antibodies, improve antitumor efficacy and prolong overall survival time in patients with many solid tumors, including lung cancer, melanoma, gastrointestinal cancer and so on (6–9). Though immune checkpoint inhibitors mark the arrival of a new era of cancer immunotherapy, using them alone has limited effect, for many patients encounter primary resistance or initial responses but eventually becoming resistant (10–12). Therefore, there is an urgent need to combine other treatments with ICIs to improve the therapeutic efficacy and prolong overall survival.

Immune checkpoint inhibitors produce antitumor effects through eliminating immune inhibition, recovering or even enhancing hosts’ immunity. The process of immune responses includes capturing, presenting, recognizing targets on tumor cells and finally killing tumor cells. Among so many targets on tumor cells, neoantigens are ideal ones to activate the immune system. Moreover, the neoantigen-specific CD8+ T cell reactivity plays a core role in immunotherapy (13). With observations that the absence of pre-existing immunity or the inhibition of tumor microenvironment may lead to invalidation of both methods (10, 14, 15), there is a strong rationale for combining ICIs with neoantigen vaccines (16). On one hand, adding neoantigen vaccine to ICIs can improve response rates of “hot” tumors through broadening cytotoxic T cell repertoire, as well as turn “cold” tumors to “hot” ones, therefore widening the scope of population who can benefit from immunotherapy (17–26). On the other hand, ICIs can unleash immunity to facilitate the efficacy of neoantigen vaccine. In this review, we focus on the safety and efficacy of personalized cancer vaccines combined with ICIs for the treatment of several malignancies. We highlight the recent development, challenges and possible improvements of personalized cancer vaccines in combination with ICIs, and hope to provide theoretical foundations for the development and application of personalized cancer vaccines in clinical settings.

## Rationale for Combination Immunotherapy

To better understand the mechanism of combination immunotherapy, it is necessary to learn the dynamics of anti-tumor immune responses. Researchers propose a concept called ‘‘Cancer-Immunity Cycle’’, which illustrate crucial points during anti-tumor response and consist of seven steps (27). First step, dying tumor cells release tumor antigens such as CRT, HSPs, HMGB1 and ATP. Insufficient tumor antigens release may hamper the proceeding of this cycle. Second step, immature dendritic cells (DC) capture these antigens *via* signals such as CD92, TLR4 and P2RX7, which can bind to CRT, HMGB1 and ATP, respectively. Then the DCs maturate and migrate to draining lymph nodes. Inadequate activation of DCs may halt the process. Third step, DCs will process the captured tumor antigens and present them to prime and activate effector T cells. In this process, captured antigens with MHC class I and II molecules and DC co-stimulatory signals are required to stimulate T cells. However, some factors may affect T cell priming and activation, including defective expression of MHC molecules in tumors, over-expression of inhibitory signals (CTLA4/CD80, 86, PD-1/PD-L1), limited T cell repertoire (central tolerance), suppressive cells such as regulatory T cells (Tregs). Fourth and fifth steps, the activated effector T cells traffic to and infiltrate into tumors. Sixth and final steps, these cytotoxic T lymphocytes recognize *via* MHC/peptide complexes on the cell surface and kill their target cells. Many inhibitory mechanisms are active within tumor microenvironment, such as lack of MHC molecules in tumors, increased inhibitory signals (PD-1/PD-L1, Tim-3/phospholipids, BTLA, LAG3, IDO, Arginase), Tregs, myeloid-derived suppressor cells, M2 macrophages and hypoxia. When eventually killing tumor cells, they also release additional tumor antigens to provoke further Cancer-Immunity Cycle. This secondary immunity increasing the repertoire of tumor antigens is recognized as “antigen spreading” with increased breadth and depth of anti-tumor immunity (28). However, tumors are clever, which can take various strategies to attenuate the efficiency of anti-tumor immunity, resulting in incompetent immunity in a process designated as “cancer immune-editing” (29, 30). Therefore, the Cancer-Immunity Cycle is broken. Given the complicated immunity network, it is reasonable to combine appropriate immunotherapeutic strategies to pave the way and promote the Cancer-Immunity Cycle forward. For instance, neoantigen vaccines can bypass the first two steps and directly initiate an immune cycle. Immune checkpoint inhibitors can help overcome immune-suppression in steps 3 and 6. On the basis of cancer immunity cycle and recent studies (25, 31–38), we conclude the main mechanisms for combination immunotherapy are as follows.

### Improve Sensitivity and Efficacy of Immune Checkpoint Inhibitors

Many patients with cancer initially do not response to anti-PD-1 inhibitors, possibly because of “cold” tumors, which are with no or few immune cells in tumor tissues and insensitive to ICIs (10, 39–41). In these tumors, tumor antigen cannot effectively prime and activate T cells, and further lead to the cycle halting at step 1 or 2. Vaccination with neoantigens bypassing initial two steps can produce many neoepitope-specific T cells which can traffic to tumor microenvironment and destroy tumor cells expressing these antigens. For instance, neoantigen-specific T cells were found in periphery blood after vaccination (10, 42–44). Furthermore, this strategy produces CD8+ neoantigen-specific T cells and memory T cells, and broadens the TCR repertoire of T cells, intensifying steps 3 and 4, which can further lead to tumor regression (16). Meanwhile, other studies have shown that clonally expanding neoantigen-reactive cells within tumor infiltrating lymphocytes (TILs) expressed PD-1 orPD-L1, suggesting that neoantigen vaccines could create a proper setting for ICIs and lay a foundation for the combination (31, 45). In conclusion, a potent personalized cancer vaccine with strong immunogenicity, can diversify the tumor-specific T cell repertoire, activate immune systems, activate robust effective T cells responses, and enhance the efficacy of ICIs.

### Overcome Acquired Resistance of Immune Checkpoint Inhibitors

Effective ICIs result in immune-editing, which will result in subpopulation change, depletion of neoantigens, T cells expansion constraint and finally resistance to ICIs (46–49). Neoantigen repertoire variation can also lead to acquired resistance to ICIs (50). Immune-editing and variation of neoantigen repertoire are involved in many steps of the immunity cycle. In a study, patients with non-small cell lung cancer, who became resistant to ICIs after initial response, experienced the evolution of tumor neoantigens. Neoantigens, which were targets of initial response to ICIs, were eliminated in this process (50). Meanwhile, another study showed that loss of neoantigens could deter the specific T cells expansion (51). However, in the study, two patients with recurrent tumor lesions and resistant to ipilimumab, had tumor regression after injecting neoantigen vaccines (52). Researchers have tried to explore the influence of neoantigen cancer vaccines on neoantigen-specific T cell receptor repertoire, and they found that vaccination with neoantigens could elevate TCR-β clonotypes (52). What’s more, neoantigen vaccines, which can provide strong immunogenicity to activate immune system based on the neoantigen variation spectrum, eventually overcome the acquired resistance (50). Therefore, identifying neoantigens in the tumor evolution and listing them as targets of personalized cancer vaccines can improve antitumor efficacy of ICIs in patient with resistance. Neoantigen vaccines not only speed the proceeding of the immunity cycle, but also augment some crucial points in the activity such as steps 3 and 4.

### Immune Checkpoint Inhibitors Overcome Suppressive Microenvironment

The suppression of tumor immune microenvironment is the main reason for the failure of neoantigen vaccine alone to control tumor. Some researchers found that after the neoantigen vaccine was applied, the expression of PD-1 on neoantigen-specific T cells and PD-L1 on tumor cells increased. Meanwhile, compared to CD8+ TILs, neoantigen-specific TILs displayed a more exhausted-like phenotype, which indicated that neoantigen vaccines could contribute to the inhibitory immune microenvironment (1, 13, 53–55). Both conditions weaken the potency of steps 3, 6 and 7. Some researchers tried to investigate the effectiveness of ICIs to overcome the immune-suppression and prove that ICIs could diminish the suppression of immune system and help induce strong T cells targeting at neoantigen epitopes (18). Schumacher and colleagues conducted some studies and indicated that adding ICIs to the treatment of neoantigen vaccine could elevate neoantigen T-cell response (18). In another study, in one out of three melanoma patients who have a relapse and distant metastasis after vaccination with neoantigens, a complete response was observed by subsequent pembrolizumab treatment (53). ICIs can mitigate the impact of inhibitory factors such as PD-1, PD-L1 and CTLA-4 to the Cancer-Immunity Cycle. The general hypothesis is that ICIs may unleash neoantigens with less immunogenicity or reactivate T cells with exhausting phenotypes to enhance antitumor effects (13, 24, 56–59). Increasing studies certify that ICIs can relieve immune inhibition in neoantigen vaccine, and have a promising prospect (50, 60).

## Preclinical and Clinical Trials and Recent Development

A personalized cancer vaccine, when combined with the ICIs, has shown efficacy in many preclinical trials. Meanwhile, there are many finished and on-going clinical trials trying to further prove the efficacy of the combination in real world.

### Recent Preclinical Trials of the Combinatory Modality

In the aggressive glioblastoma CT2A murine model, researchers generated the neoantigen vaccine comprising 27-mer peptides targeting the mutant Plin2G332R, Pomgnt1R497L, and Epb4H471L neoepitopes, as well as poly-ICLC adjuvant. Mice treated either with vaccine or anti-PD-L1 alone exhibited a median overall survival of 17.5 and 25 days, respectively. In contrast, 60% of mice treated with vaccine and anti-PD-L1 blockade demonstrated long-term survival. What’s more, tumor-infiltrating neoepitope-specific CD8 T cells increased in the combinatory condition (25). In five murine colon carcinoma models, researchers investigated the clinical efficacy of the combining a peptide or DNA vaccine with anti-PD-1/L1 and anti-CTLA-4. The neoantigen vaccines combined with immune checkpoint inhibitors can potentiate neoantigen-specific immunity, elicit robust and long-lived T-cell response with a more diversified TCR repertoire and potentially inhibit even eradicate tumors without re-challenge (16, 49, 61–63). Furthermore, three studies have initially proven the central role of CD8+ T cells in the combination regimen by depletion of CD8+ T cells, CD4+ T cells and natural killer 1.1, respectively (16, 49, 63). Besides, vaccines with nanocomplexes markedly improve Ag/adjuvant co-delivery to lymphoid organs and sustain Ag presentation on dendritic cells (61–64). In two highly aggressive and poorly immunogenic murine models of B16F10 melanoma, researchers obtain the similar result that neoantigen vaccine combined with ICIs can initiate potent anti-tumor efficacy (62, 64). Additionally, Kuai et al., also reported vaccines administered *via* the subcutaneous (SC) or intramuscular (IM) routes were well tolerated in mice without any significant systemic or local toxicity, whereas SC could more efficiently deliver vaccines and intensify neoantigen-specific T cells responses (64). Finally, Panc02 cells models provide proof of concept that triple therapy with PancVAX (a personalized cancer vaccine), anti-PD-1, and agonist OX40 induces vaccine-specific TILs, lower the threshold for T cell activation, and reducing TIL exhaustion markers such as LAG3 and PD-1. In KPC mice (with KRAS and p53 mutations, pancreatic ductal adenocarcinoma), a “cold” tumor, combination treatment can also elicit objective tumor responses and prolonged survival. More importantly, this study shows that sequential combination treatment, neoantigen vaccine prior to anti-PD-1 antibodies significantly increased INF-γ expression and cure rates compared to single regimen or in combination with anti-PD-1 blockadeconcurrently (65, 66). What’s more, in a hepatic cell cancer model, similar results are reported (67). These preclinical trials are displayed in **Table 1**.

**Table 1:** Preclinical trials of neoantigen vaccines combined with immune checkpoint inhibitors.

Type of neoantigen vaccine	Formulation	Type of tumor	Immune checkpoint inhibitor	Antitumor effects comparing to vaccine or ICIs alone	Route of vaccination	Ref
Peptide	Albumin/AlbiAdpgk nanocomplexes	Colon cancer(MC38 tumor)	Anti-PD-1	More effective	SC	(61)
Peptide	sHDL-Adpgk and adjuvants	Colon cancer and melanoma (MC-38 and B16F10 tumors)	Anti-PD-1, anti-CTLA-4	More effective	SC	(62)
Peptide	sHDL-Adpgk/CpG	Advanced B16F10 melanoma tumors	Anti-PD-1, anti-CTLA-4	More effective	SC or IM	(64)
Peptide	PancVAX	Pancreatic adenocarcinoma (Panc02 cells)	Anti–PD-1	More effective	SC	(65)
DNA	poly-neoantigen DNA vaccine	Colorectal tumor MC38	Anti-PD-1	More effective	Intradermal injection	(49)
DNA/mRNA	GAdCT26-31/GAd-MC38-7	Colon carcinoma (CT26 cell line, MC38 cell line)	Anti-PD1, anti-PDL1	More effective	SC	(16)
Peptide	Lm-ANXA2	Pancreatic carcinoma	Anti-PD1	More effective	Injection directly through the spleen	(66)
Peptide	Adpgk with adjuvants (banNVs)	Colorectal cancer	Anti-PD-1	More effective	SC	(63)
Peptide	Multivalent neoantigen vaccine	Glioblastoma (CT2A GBM model)	Anti–PD-L1	More effective	None	(25)
Peptide	Thiolated nano-vaccine	Hepatocellular carcinoma(H22 cells)	Anti–PD-1	More effective	SC	(67)

SC, subcutaneous; IM, intramuscular; GAdCT26-31/GAd-MC38-7, vaccines of great ape adenovirus encoding multiple neoantigens; Lm-ANXA2m listeria-based, ANXA2-targeting vaccines; Adpgk, ADP dependent glucokinase; CpG, cytidine-phosphate-guanosine; sHDL, synthetic high-density lipoprotein; PancVAX, neoantigen-targeted vaccine.

### Recent Completed and Ongoing Clinical Trials

The first open-label phase IB clinical trial (NCT02897765) of a personalized neoantigen-based vaccine, NEO-PV-01, in combination with PD-1 blockade, included 82 patients with advanced solid tumors. Analyzing 82 patients, the median progress free survival (PFS) among vaccinated patients was 23.5, 8.5, and 5.8 months in the melanoma, NSCLC, and bladder cancer cohorts, respectively. The median OS for vaccinated patients was not reached in the melanoma and NSCLC cohorts, while for the bladder cancer cohort, the median OS was 20.7 months. The primary objective of the study was to evaluate the safety and tolerability of NEO-PV-01 in combination with nivolumab. The most common adverse events in vaccinated patients were injection-site reactions and influenza-like illness (52 and 35% of the patients, respectively). No treatment-related serious adverse events were observed. These data support the safety and immunity of this regimen in patients with advanced solid tumors (68).

A phase IB study (NCT03289962) evaluated RO7198457, an individualized neoantigen-specific Immunotherapy (iNeST), in combination with atezolizumab in 144 patients with locally advanced or metastatic solid tumors. RO7198457 is a kind of mRNA vaccine including up to 20 neoantigens. In 108 analyzable patients, nine patients responded to the therapy (ORR 8%) including one complete response (CR) and 54 patients (49%) experienced stable disease (SD). The vaccine induced neoantigen-specific T cell response in 77% patients. The combination was well tolerated, and most adverse events were infusion-related reaction, fatigue, cytokine release syndrome, nausea, pyrexia in over 10% patients, classified as grade 1 or 2. No increase in immune-mediated AEs compared with anti-PD-1 alone. Results prove that the combination of RO7198457and atezolizumab is safe and effective, which can induce significant neoantigen-specific immune response (69).

Another phase I multicenter study (NCT03313778) is to assess the safety, tolerability, and immunogenicity of mRNA-4157 alone in 13 patients with resected solid tumors and in combination with pembrolizumab in 20 patients with unresectable solid tumors. Indications include melanoma, NSCLC, MSI-high CRC, metastatic cutaneous squamous cell cancer, bladder cancer and so on. mRNA-4157, is a lipid-encapsulated RNA-based neoantigen-based vaccine. Of the 13 patients, 12 patients remain disease free on study with median follow-up of 8 months. While in another 20 patients, researchers observed one CR, two partial responses (PR), five SD, five progressive diseases (PD), two introduced immune unconfirmed progressive disease, and one patient non-evaluable for response. No dose of limited toxicity (DLT) and no drug related SAEs or AEs ≥ grade 3were reported, and treatment related AEs have generally been of low grade and reversible. These results also demonstrate the antitumor efficacy and safety of the combination with pembrolizumab and neoantigen-specific T cells, proceeding mRNA-4157 to phase2 (70, 71).

ADXS-NEO-02 is an ongoing Phase 1 trial (NCT03265080), which preliminarily investigates the safety and efficacy of ADXSNEO alone and in combination with anti-PD-1 antibody therapy in solid tumors. ADXS-NEO is composed of the listeriolysin O(tLLO) and personalized tumor antigens with 20–21mer peptides. This initial result reveals the optimal dose of ADXS-NEO with safety and efficacy. Only mild/moderate, controlled, and reversible events (e.g., chills, fever, tachycardia) were observed at this level. Efficient and rapid priming of substantial CD8+ T cells against most neoantigens and increased secretion of chemokines consistent with T-cell trafficking into tumor microenvironment also existed. Further investigation results about the combination of ADXS-NEO + anti-PD-1 antibody therapy are waiting (72).

Another ongoing phase I/IIA study (NCT03633110), is exploring the tolerability and antitumor activity of GEN-009 combined with anti-PD-1/L1 in multiple advanced tumors. GEN-009 is a personalized neoantigen-based vaccine comprising 4–20 synthetic long peptides formulated with poly-ICLC. The preliminary study of GEN-009 alone in solid tumors displayed that neoantigen provoked sustained peripheral neoantigen-specific CD4+ T cell and CD8+responses in all eight patients. Besides, repeated dosing has been well tolerated with mild local discomfort and no DLT. Further data from this trial about the vaccine in combination with PD-1 inhibitors in patients with advanced tumors, are awaited (71, 73).

Another open-label, phase IB study (NCT03380871) of NEO-PV-01 with pembrolizumab plus chemotherapy in patients with advanced or metastatic non-squamous non-small cell lung cancer is completed, and the results are expected. More clinical trials combining personalized vaccines with immune checkpoint inhibitors are listed in **Table 2**.

**Table 2:** Recent clinical trials combining neoantigen vaccines with immune checkpoint inhibitors.

ClinicalTrial.gov identifier	Phase	Enrollment status	Cancer Type	Neoantigen Formulation	Additional intervention	Delivery	Dose and Schedule
NCT04397003	II	Not yet recruiting	ES-SCLC	Neoantigen DNA	Anti-PD-L1(durvalumabMEDI4736)	Intramuscular by electroporation	Not specified, six q4w cycles
NCT03897881	II	Recruiting	M	Neoantigen mRNA-4157	Anti-PD-1(pembrolizumab)	None	1,000 mg nine q3w cycles
NCT04267237	II	Withdrawn	NSCLC	Neoantigen mRNA(RO7198457)	Anti- PD-L1(atezolizumab)	Intravenous infusion	Not specified, 12 q4w cycles
NCT03815058	II	Recruiting	Advanced M	Neoantigen mRNA(RO7198457)	Anti-PD-1(pembrolizumab)	None	Not specified; qw priming and booster
NCT03606967	II	Recruiting	Metastatic TNBC	poly-ICLC+ Neoanitgen synthetic long peptides	Anti-PD-L1 (durvalumabMEDI4736)	Subcutaneous	None
NCT03598816	II	Withdrawn	RCC	Neoantigen DNA	anti-PD-L1 (durvalumab]) anti-CTLA-4 (tremelimumab)	Intramuscular	six doses
NCT03953235	I, II	Recruiting	NSCLC, CRC, PC, other solid tumors	Neoantigen Peptides (GRT-C903 and GRT-R904)	anti-PD-1(Nivolumab)and anti-CTLA-4(ipilimumab)	None	None
NCT03639714	I, II	Recruiting	NSCLC, MSSCRC, EC, BC	Neoantigen adenovirus vector + self-amplifying mRNA(GRT-C901 and GRT-R902)	anti-PD-1(nivolumab) and anti-CTLA-4(ipilimumab)	Intramuscular *via* viral vector	30–300 mg, Priming and booster
NCT04251117	I/IIA	Recruiting	HCC	Neoantigen DNA vaccine (GNOS-PV02)	Anti-PD-1(pembrolizumabMK-3475)	Intradermal injection and electroporation	None
NCT03164772	I, II	Active, not recruiting	NSCLC	mRNA Vaccine (BI 1361849, CV9202)	anti-PD-L1 (durvalumab) + anti-CTLA-4 (tremelimumab)	None	None
NCT04024878	I	Recruiting	OC	Poly-ICLC+Neoantigen peptide (NeoVax)	Anti-PD-1(nivolumab)	Injection underneath the skin	Not specified; priming and booster
NCT02897765	I	Completed	UBC, BT, T CCB,MM, M,SC, NSCL	Neoantigen peptides(NEO-PV-01)	Anti-PD-1(nivolumab)	Subcutaneous	Not specified
NCT03289962	I	Recruiting	Solid Cancers	Neoantigen mRNA (RO7198457)	Anti-PD-L1(atezolizumab)	Intravenous infusion RNA-lipoplex,	25–100 mg qw priming and boosters
NCT03568058	I	Active, not recruiting	Advanced Cancer	Neoantigen peptide	Anti-PD-1(pembrolizumab)	None	None
NCT04266730	I	Not yet recruiting	NSCLC,SCCHN	Neoantigen peptide vaccine (PANDA-VAC) + Poly-ICLC	Anti-PD-1(pembrolizumab)	Subcutaneous	1,800 ug, 2,400 ug, priming and booster
NCT04161755	I	Recruiting	PC	Neoantigen mRNA(RO7198457)	Anti-PD-L1(atezolizumab)	None	None
NCT04072900	I	Recruiting	M (Skin)	Neoantigen peptide	Anti-PD-1(toripalimab)	None	4 × 3 mg all the peptides given by seven times
NCT03597282	I	terminated	Metastatic M	Poly-ICLC + neoantigen peptides(NEO-PV-01)	Anti-PD-1(nivolumab),	Subcutaneous	None
NCT02287428	I	Recruiting	GB	Neoanitgen peptide(NeoVax)	Anti-PD-1(pembrolizumab)	None	Not specific, priming and boost phases.
NCT04799431	I	Not yet recruiting	PC,MCRC	Neoantigen Vaccine + Poly-ICLC	Anti-PD-1(retifanlimab)	Subcutaneous	0.3 mg per peptide vaccine
NCT04248569	I	Recruiting	FLC	Neoanitgen peptide(DNAJB1-PRKACA fusion kinase)	Anti-PD-1(nivolumab)and anti-CTLA-4(ipilimumab)	None	Not specifically, Priming and booster
NCT03219450	I	Not yet recruiting	LL	Neoanitgen peptide(NeoVax)+ Poly-ICLC	Anti-PD-1(pembrolizumab)	None	Not specifically, priming and booster
NCT03121677	I	Recruiting	FL	Personalized tumor vaccine+ Poly-ICLC	Anti-PD-1(Nivolumab)	Subcutaneous	Not specifically
NCT03166254	I	Withdrawn	NSCLC	Neoantigen peptides(NEO-PV-01)+ Poly-ICLC	Anti-PD-1(pembrolizumab)	None	Not specifically, priming and booster
NCT03199040	I	Recruiting	TNBC	Neoantigen DNA	Anti-PD-L1 (durvalumab)	None	None
NCT03532217	I	Active, not recruiting	MHSPC	Neoantigen DNA	Anti-PD-1(nivolumab)and anti-CTLA-4(ipilimumab)	Intramuscular	4 mg, priming and booster
NCT03380871	I b	Completed	Advanced or metastatic NSCLC	Neoantigen peptides(NEO-PV-01)+ Poly-ICLC	Anti-PD-1(pembrolizumab)	Subcutaneous	None
NCT02950766	I	Recruiting	High-risk RCC	Neoanitgen peptide(NeoVax)+ Poly-ICLC	Anti-CTLA-4(ipilimumab)	Subcutaneous	Not specifically, priming and booster
NCT03359239	I	Recruiting	UC/BC	Neoanitgen peptide (PGV001) + Poly-ICLC	Anti-PD-L1(atezolizumab)	None	Up to ten synthetic peptides—100 μg (0.01 ml, 10 mg/ml) per peptide. One tetanus helper peptide—100 μg (0.01 ml, 10 mg/ml),up to ten total doses
NCT03422094	I	Terminated	GBM	Neoanitgen peptide(NeoVax)+ Poly-ICLC	Anti-PD-1(nivolumab)and anti-CTLA-4(ipilimumab)	Subcutaneous	Not specifically, priming and booster
NCT03929029	Ib	Recruiting	M	Neoanitgen peptide(NeoVax)+ Poly-ICLC+ Montanide	Anti-PD-1(nivolumab)and anti-CTLA-4(ipilimumab)	None	None
NCT04117087	I	Recruiting	CRC, PC	KRAS peptide+ Poly-ICLC	Anti-PD-1(nivolumab)and anti-CTLA-4(ipilimumab)	None	1.8 mg, priming and booster

ES-SCLC, extensive stage small cell lung cancer; NSCLC, non-small cell lung cancer; MCRC, metastatic colorectal cancer; CRC, colorectal cancer; MSSCR, microsatellite state colorectal cancer; UBC, urinary bladder Cancer; UC/BC, urinary/bladder carcinoma; BT, bladder tumors; TCCB, transitional cell carcinoma of the bladder; MM, malignant melanoma; M, melanoma; SC, skin cancer; SCCHN, squamous cell carcinoma of head and neck; HCC, hepatic cell cancer; TNBC, triple negative breast neoplasms; RCC, renal cell carcinoma; LL, lymphocytic leukemia; FL, follicular lymphoma; PC, pancreatic carcinoma; MHSPC, metastatic hormone-sensitive prostate cancer; FLC, fibrolamellar hepatocellular carcinoma; OC, ovarian cancer; GBM, glioblastoma; Poly-ICLC, polyinosinic–polycytidylic acid-poly-l-lysine carboxymethylcellulose.

## Safety and Toxicity Associated with the Combination Modality

The application of ICI therapy comes with the possibility of occurring immune related adverse events (IRAE). IRAE are regarded as an “over-activation” of the immune system leading to autoimmune inflammatory events affecting virtually any organ, most commonly the skin, gastrointestinal tract, liver, endocrine system and lung (74–76). In this review, we have highlighted the combination of neoantigen vaccine with ICI. Neoantigen vaccine and ICI both effect *via* provoking the immune system. Consequently, there is a relevant concern that the combination may lead to excessive toxicity. Overall, clinical experience with the combination strategies discussed in this review is limited. However, in a phase I trial (NCT02897765 n = 82) evaluating the safety and tolerability of NEO-PV-01 in combination with nivolumab, injection-site reactions (52%) and influenza-like illness (35%) are most common. Injection site reactions such as warmth and erythema, were often reversible and mild (Common Terminology Criteria for Adverse Events [CTCAE] grade 1), except one patient with a grade 2 injection site erythema. Drug-related events of grade ≥3 severity appeared in two patients. One patient with grade 2 gastritis discontinued the treatment. There is no treatment-related serious adverse events (68). In another phase Ib study (NCT03289962 n=142) to assess RO7198457 in combination with atezolizumab, immune-mediated adverse events (AEs) in atezolizumab alone were similar to those of the combination in >10% of patients include infusion-related reaction (60%), cytokine release syndrome (15%), influenza-like illness (10%), fatigue (30%), nausea (22%), pyrexia (15%), diarrhea (19%), decreased appetite (15%), vomiting (14%), headache (12%), cough (15%), dyspnea (15%), arthralgia (10%), constipation (15%),anemia (12%). Individual signs and symptoms of systemic reaction in more than five patients involve pyrexia, chills, nausea, tachycardia, headache, vomiting, hypertension, myalgia, back pain, fatigue, and hypoxia. 1% patients had grade 3 infusion-related reactions, anemia, fatigue, dyspnea, vomiting, nausea, pyrexia, diarrhea, headache, respectively. Most AEs are grade 1 or 2, and systemic reactions were transient and generally manageable in the outpatient setting. No grade 4 or 5 AE was observed (69). InmRNA-4157 trial, there was also no serious treatment related AEs, with only low grade and reversible reactions. Generally speaking, initial results of studies prove the treatment regimen of neoantigen vaccine combined with ICIs efficient and safe. Whereas, the actual risk for severe adverse events with combinations and potential factors influencing safety such as dose, delivery platforms, ways of administration, adjuvant, personal status, will be required to more and larger randomized studies.

## Main Considerations Relating to Therapeutic Combinatory Regimen

Various factors may affect the anti-tumor efficacy in the combinatory modality. For neoantigen vaccine, neoantigen selection is critical, which will determine the production of antigen-specific T cells and eventually influence the anti-tumor response. The pipeline for neoantigen identification includes five main steps, and details have been extensively reviewed in other reviews (77–79). Unfortunately, there is no perfect method to predict appropriate neoantigen without bias and negatively positive. What’s more, the selection of proper delivery platforms is also crucial. Seven kinds of vectors are explored, including synthetic peptides, messenger RNA, DNA plasmids, viral vectors (adenoviral and vaccinia), engineered attenuated bacterial vectors (Salmonella, Listeria), *e x vivo* antigen-loaded DCs, and nanodiscs. Their advantages and disadvantages have been listed in detail in previous reviews (80, 81). The central problems are the delivery efficacy and manufacturing time, which play an important role in initiating enhanced T cell responses to inhibitor tumors. The sequence of manufacturing time from shorter to long is approximately tumor lysate-pulsed DCs, DNA/RNA, peptide, neoantigen-pulsed DC (71). For both peptide and mRNA vaccine platforms, time less than 4 weeks is expected (80). Additional variables include the route of administration of the vaccine, total number of doses, and induction (priming) and booster (maintenance) intervals. In a preclinical trial, Rui Kuai et al. declared that subcutaneous injection, comparing with intramuscular way, have stronger capability to deliver vaccines and provoke neoantigen-specific T cells responses (64). With the dose increasing, the immune response is intensified and the occurrence of immune related adverse events also elevate. The key point is to find the balance between efficacy and safety (82–87). Owing to lack of more clinical data, further studies about dose and administrating route are urgently required (64). When it comes to the combination, a vital problem emerges, the sequence of administration of neoantigen vaccines and ICIs. Pre-vaccination promote baseline immunity, significantly increase expression of INF-γ, Fms-related tyrosine kinase 3 ligand (FLT3L) and granulocyte–macrophage colony-stimulating factor(GM-CSF), and help “cold” tumor, such as glioblastoma and pancreatic ductal carcinoma, respond to ICIs (65, 66, 71). Additionally, administration of ICIs at vaccination or post-vaccination can boost vaccine-induced immune response. Unfortunately, limited data is presented, so further endeavors are necessary to explore the proper sequence of administration. To monitoring antitumor efficacy, applications of gene sequencing and single-cell sequencing technologies might find why resistance appear and discover the alternative neoantigens.

## Challenges and Improvements

Though the combination of neoantigen vaccines and ICIs is promising in personalized treatment, there are still many problems.

### Selection of Population Who May Potentially Benefit From the Combination

ICIs and neoantigen vaccines alone can only benefit a fraction of patients, efforts to find out the most proper population to receive the combination therapy are urgently required (88).

Recent studies have shown that somatic mutation, neoantigen burden and neoantigen density associated with long-term benefit from immune checkpoint inhibitors in several solid tumors, which indicated that more mutation-associated neoantigens may enhance the immunogenicity and improve immune responses of ICIs (19, 44, 89). The possible explanation is that higher mutational burden provides a base to generate more immunogenic neoantigens. Furthermore, researchers have found there were neoantigen-specific T cells in the peripheral blood in patients with tumor regression, and this also demonstrated that some neoantigens indeed could activate T cell which had an antitumor effect (19, 44, 88, 90). Therefore, neoantigen burden may be a significant predictor for combinational immunotherapy to distinguishing responders from non-responders.

However, some studies also revealed that responders to ICIs were not restricted to patients with high neoantigen burden, which demonstrated that not only the quantity of neoantigens is significant, rather their “quality” is vital as well (91, 92). For example, in melanoma, an effective antitumor CD8+ T cells response can be produced by a few epitopes, which have affinity to TCRs (26, 43). Another instance, renal cell carcinoma (RCC) has only a moderate to low mutation rate; however, it is universally known that RCC has a good response to immunotherapy (93). Researchers tried to explain this phenomenon and discovered that RCC possess the highest level of insertion and deletion type (indel) mutations, which are regarded to frequently create a new open reading frame and a higher proportion of neoantigens (94). Considering the quality of mutation into the prediction is also significant.

What’s more, lower neoantigen intra-tumor heterogeneity (ITH) is correlated with significantly longer progression-free survival. Neoantigen heterogeneity has a high variable rate with an average of 44% neoantigens found heterogeneously, in a subset of tumor regions (range of 10 to 78%) (55). Researchers have observed that lower hazard ratios when considering both neoantigen burden and ITH compared with the use of neoantigen burden alone to screen the potential population (55). Additionally, tumors with low ITH had an elevated PD-L1 expression (55). Thus, combining heterogeneity selecting a proper threshold with neoantigen burden is critical to find out the population who may benefit from ICIs and neoantigen vaccines.

Effectively controlling tumors with personalized vaccines and ICIs, is associated with neoantigen-specific T cells, which can exist in peripheral blood and show a phenotype different from patients without a response. Further studies to investigate characterization of neoantigen-specific T cells in responder patients, showed that there was relatively high expression of the activation markers CD161, TIGIT, 2B4 and KLRG1,low level of expression of inhibition markers CD27, CD28 and CD127, and a high level of co-inhibitory molecules, including PD-1 (95, 96). These results indicate that identification and characterization of neoantigen-specific T cells may contribute to predict who will response to the combination therapy.

### Neoantigens Heterogeneity and Dynamic Variation of Neoantigen Landscape

Neoantigens arise from tumor-specific mutations, and they are variable in different tumors or patients. ITH has a significant impact on the response to immunotherapies. In addition, in patients who initially respond to ICIs, tumors could experience an evolution of epitopes, which will alter the neoantigen landscape and lead to sequential resistance. More importantly, mechanisms of ITH and the dynamic variation of neoantigens landscape are unclear for now (13, 97, 98). We just observed that high heterogeneity may lead to a poorer response to both vaccination and ICIs. For this problem, researchers provide that designing a neoantigen vaccine with multiple targets and based on the variation landscape may help overcome ITH and the dynamic alteration (55).

### Identification of Potential Neoantigens

Exact identification of the neoantigens is capable of producing potent epitopes which can be recognized specifically by TCRs and induce strong immune response. However, this is difficult to achieve in current silico predicting systems (49). Some studies showed that in predicted neoantigens, only a fraction (20%) had immunogenicity, demonstrating the low accuracy of current neoantigens prediction algorithms (99, 100). For now, there is no single method providing an accurate and reliable prediction and identification of neoantigens. The possible solutions for this problem are as following. One method is designing multi-epitope vaccines. Using of a platform which can accommodate a large number of neoantigens, may help overcome limits of the prediction algorithms. In a study, combing treatment with the adenoviral vaccine targeting 31 neoantigens increased the number of mice with complete regression (~50%), which may be due to the increasing specific T-cell clones (8). These findings demonstrate that multi-epitope vaccines may be a solution for inaccuracy of the bioinformatics tools for predicting neoantigens (8). Another option is to broaden our analysis of potential neoantigens to include other types of potentially immunogenic alterations, for current predicted neoantigens are mainly resulting from missense mutations (2). For example, chromosomal insertions, inversions, and translocations can lead to fusion transcripts, which exist in certain cancers, such as chronic myelogenous leukemia, lung cancer, bladder cancer, and ovarian cancer (101, 102).

### Faster and Cost-Effective Vaccine Production

Currently, although the emergence of NGS, WES and other techniques improve the identification and production of neoantigen vaccines, verifying neoantigens and vaccines generation are still time-consuming and expensive, and usually 3–5 months are required to prepare vaccines from tumor samples (26, 43). Additionally, problems that the identification of neoantigens needs lots of tumor tissues, while the yield of usable epitopes or neoantigens is very low, and it is difficult to solve for technical limits (103). These obstacles have been largely hampering the development of neoantigen vaccines in clinical settings. There is an urgent need to develop better neoantigen prediction algorithms and manufacturing technologies which can decrease the price and shorten the time (4, 104). Efforts for this work are going on, and Anna have established a fast process assembling 60 unique patient mutanome-specific neoantigens and producing personalized adenoviral vaccines within 6 weeks from the time of patient biopsy (16).

### Safe and Efficient Delivery System

To generate a potent neoantigen vaccine, a safe and efficient delivery system is needed, which can help induce strong immune response. Effector T cells can be induced by the specific antigens or epitopes within the tumor cells and neoantigen-specific T cells clonal expansion in tumors symbols the effective antitumor response. Neoantigen vaccines based on virus could be the proper candidates to produce potent antitumor immunity. Many studies have proven adenoviruses were powerful genetic vaccine platforms with unique feature encoding for large antigen to activate effective CD8+ and CD4+ T-cell responses safely (105–107). In a preclinical study, a adenoviral vaccine encoding 31 neoantigens, the largest number used so far for neoantigen-based vaccines, selected from the murine CT26 colon carcinoma cell line, produced potent antitumor immune responses, and more than 1,000 antigen-specific IFN-γ secreting lymphocytes/million splenocytes, CD8+ and CD4+ T lymphocytes were generated (16).

### Low Efficacy of Neoantigen Vaccines

In preclinical trials, researchers did not detect neoantigen-specific T cells in untreated mice with tumors. The possible reasons are showed below: the inadequate mutated gene expression; antigen can’t be presented effectively due to low affinity to HLA or TCRs on T cells; dynamic variation of neoantigen landscape leading to dominant neoantigens eliminated; T cells in the repertoire which can bind to mutant neoepitopes are in short or undergoing apoptosis (65). Two approaches are capable of enhancing neoantigen-specific T cell responses and improving the antitumor potency.

One is to use a potent adjuvant to stimulate innate immunity. In some preclinical studies, researchers found that adding an agonist OX40 antibody to PancVAX, decreased T cell exhausted markers, such as Lag3 and PD-1, and helpedCD4+ T cell avoid immunosuppressive Treg phenotype (108, 109). What’s more, FoxP3+CD4+T cells decreased and tumor-specific IFN-γ-secreting CD4+ T cells appeared when combining PancVAX with OX40 (65). Furthermore, OX40 is capable of increasing the survival rate of antitumor T cells with low avidity (94). These findings suggest that an effective adjuvant can produce neoantigen-specific TILs, help activate T cells and maintain TILs through survival improvement.

Another strategy to improve the efficacy of neoantigen vaccines is taking MHC class II peptides into vaccination design (88). The clonal expansion of tumor-specific T cells with potent antitumor ability is the core of the success in the personalized cancer immunotherapy (49). Kreiter and colleagues conducted a study suggesting that many personalized cancer vaccines with immunogenic neoantigens were correlated to MHC class II molecules on CD4+ helper T cells (110). In the study, PancVAX played its role to control or shrink tumors primarily through CD8+ T cells, meanwhile, CD4+ T cells may have influence as well. Researchers further investigated the influence of both CD8+ T cells and CD4+ T cells, and noted that antitumor response disappeared when depleting CD8+ T cells while partial responses loss with CD4+ T cells eliminated. The successful example of immunotherapy in melanoma was due to existence of epitopes targeting both CD4+ and CD8+ T cells (16, 20, 26, 65). Therefore, vaccination with neoantigens targeting both CD8+ T cells and CD4+ T cells is vital to induce powerful antitumor immune responses, for the primary immune driver CD8+ T cells can work better with the synergy of CD4+ T cells (111, 112).

### Dose and Sequence of ICIs and Vaccines

Though many preclinical and clinical trials investigate the safety and efficacy of the combination of ICIs and neoantigen vaccines, dose and schedule for ICIs and vaccines have been minimally studied. Some researchers found that after the response to interferon secreted by T cells, both PD-1 on activated T cells and PD-L1 on tumors emerged in a short time, which supports that using ICIs first or concomitant with vaccines may be both rational (113). In some studies, after vaccination with neoantigens in patients, the expression of both PD-1 on neoantigen-specific T cells and PD-L1 in tumor tissues increased, and anti-PD-1 or anti-PD-L1 immunotherapy improved the efficacy of vaccines, suggesting that administering neoantigen vaccines before ICIs may have a greater opportunity to achieve the maximal antitumor response (1, 13, 20, 36, 53–55). We have searched up many preclinical and clinical trials, unfortunately, we didn’t find a standard sequence or dose for the combination, which need further exploration.

## Conclusion

Neoantigen vaccines alone have a limited efficacy, and ICIs has been limited to a minority of patients with certain cancer types. However, the combination of personalized vaccines and ICIs can significantly improve the antitumor efficacy with minimal additional toxicity compared to either single method by improving sensitivity and efficacy of ICIs, overcoming acquired resistance of ICIs and relieving suppressive microenvironment. This has led to envisaging and developing combined strategies that might augment tumor regression and prolong overall survival for patients with metastatic cancer. However, there still some important aspects for the combination to achieve the maximal efficacy, including optimizing the identification, predication and production of neoantigen vaccines, selecting proper population for the combination therapy, and the optimized dose and sequence of the two agents.

## Author Contributions

SZ contributed conception and overall idea of the study. J-YL wrote the first draft of the manuscript. SZ wrote sections of the manuscript. All authors contributed to the article and approved the submitted version.

## Conflict of Interest

The authors declare that the research was conducted in the absence of any commercial or financial relationships that could be construed as a potential conflict of interest.

## Abbreviations

CRT, calreticulin; ATP, adenosine triphosphate; HMGB1, High Mobility Group Box 1; HSPs, heat shock proteins; LN, lymph node; DCs, dendritic cells; CTLs, cytotoxic T lymphocytes; IDO, indoleamine 2,3-dioxygenase; mAb, monoclonal antibody; LAG3, Lymphocyte activation gene 3; BTLA, B and T lymphocyte associated gene.
